# Supplementary figures and images for: Diversity and Composition of Rumen Bacteria, Fungi, and Protozoa in Goats and Sheep Living in the Same High-Altitude Pasture
Source: Animals (Basel). 2020 Jan 22;10(2):186. doi: 10.3390/ani10020186 (PMC7070549; doi:10.3390/ani10020186)

Bacteria

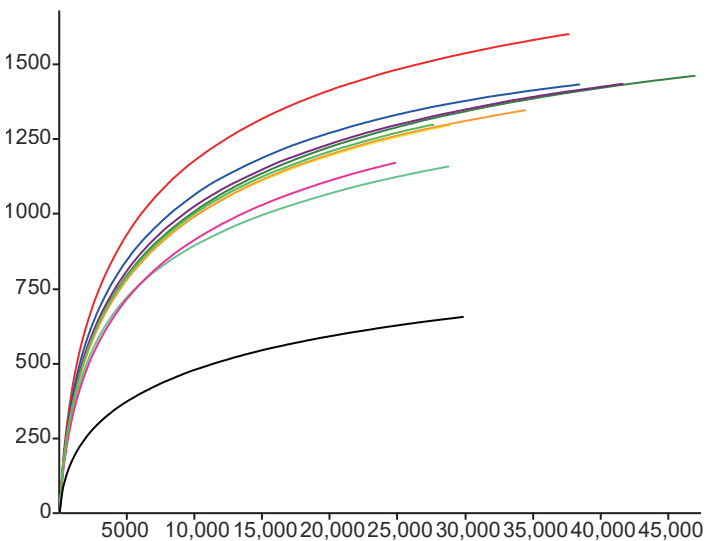

Fungi

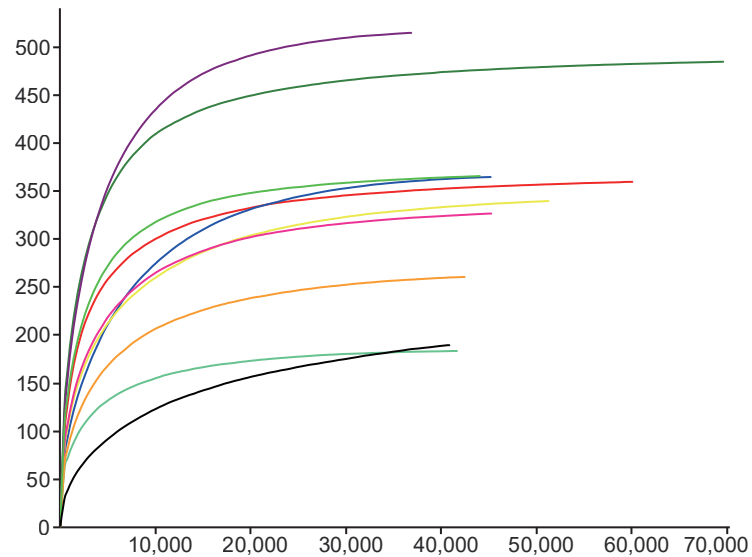

Protozoa

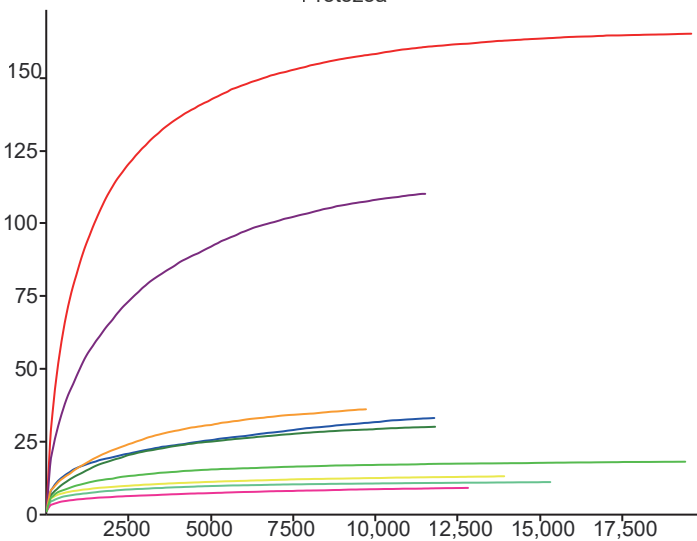

- Goats\_1
- Goats\_2
- Goats\_3
- Goats\_4
- Goats\_5
- Sheep\_1
- Sheep\_2
- Sheep\_3
- Sheep\_4
- Sheep\_5

Supplement: Supplementary file 1 [file animals-10-00186-s001.zip › Supplement Figure 1.pdf]
